# TDP-43 promotes tau accumulation and selective neurotoxicity in bigenic *Caenorhabditis elegans*

**DOI:** 10.1242/dmm.049323

**Published:** 2022-04-27

**Authors:** Caitlin S. Latimer, Jade G. Stair, Joshua C. Hincks, Heather N. Currey, Thomas D. Bird, C. Dirk Keene, Brian C. Kraemer, Nicole F. Liachko

**Affiliations:** 1 Department of Laboratory Medicine and Pathology, University of Washington, Seattle, WA 98195, USA; 2 Geriatrics Research Education and Clinical Center, Veterans Affairs Puget Sound Health Care System, Seattle, WA 98108, USA; 3 Department of Neurology, University of Washington, Seattle, WA 98104, USA; 4 Division of Medical Genetics, Department of Medicine, University of Washington, Seattle, WA 98104, USA; 5 Department of Psychiatry and Behavioral Sciences, University of Washington, Seattle, WA 98195, USA; 6 Division of Gerontology and Geriatric Medicine, Department of Medicine, University of Washington, Seattle, WA 98104, USA

**Keywords:** TDP-43, Tau, Amyloid β (Aβ), *Caenorhabditis elegans*, Alzheimer's disease, Proteotoxicity

## Abstract

Although amyloid β (Aβ) and tau aggregates define the neuropathology of Alzheimer's disease (AD), TDP-43 has recently emerged as a co-morbid pathology in more than half of patients with AD. Individuals with concomitant Aβ, tau and TDP-43 pathology experience accelerated cognitive decline and worsened brain atrophy, but the molecular mechanisms of TDP-43 neurotoxicity in AD are unknown. Synergistic interactions among Aβ, tau and TDP-43 may be responsible for worsened disease outcomes. To study the biology underlying this process, we have developed new models of protein co-morbidity using the simple animal *Caenorhabditis elegans.* We demonstrate that TDP-43 specifically enhances tau but not Aβ neurotoxicity, resulting in neuronal dysfunction, pathological tau accumulation and selective neurodegeneration. Furthermore, we find that synergism between tau and TDP-43 is rescued by loss-of-function of the robust tau modifier *sut-2*. Our results implicate enhanced tau neurotoxicity as the primary driver underlying worsened clinical and neuropathological phenotypes in AD with TDP-43 pathology, and identify cell-type specific sensitivities to co-morbid tau and TDP-43. Determining the relationship between co-morbid TDP-43 and tau is crucial to understand, and ultimately treat, mixed pathology AD.

## Abstract (precis)

**Summary:** TDP-43 co-pathology is a frequent feature of Alzheimer's disease. This paper shows that TDP-43 specifically enhances tau neurotoxicity, but not amyloid β, to promote pathological protein accumulation, behavioral impairment and selective neurodegeneration.

## INTRODUCTION

Alzheimer's disease (AD) is pathologically defined by the presence of extracellular amyloid β (Aβ) plaques and intracellular tangles of hyperphosphorylated tau (ptau) (Mirra et al., 1991; Braak and Braak, 1991; Braak et al., 2006; Thal et al., 2002; Hyman et al., 2012). Both Aβ and tau are implicated in AD neurodegeneration. Mutations in genes involved in the processing of amyloid precursor protein (APP) cause some cases of inherited familial AD, supporting a mechanistic role for Aβ pathology in the disease (Latimer et al., 2021). However, tau pathology correlates best with clinical disease progression and severity (Nelson et al., 2012; Chen and Mobley, 2019). Although mutations in the gene encoding tau have not been shown to directly cause AD, they are causal for another neurodegenerative tauopathy, frontotemporal lobar degeneration (FTLD)-tau, indicating that dysfunction of tau also plays a mechanistic role in disease (Strang et al., 2019). Numerous models of Aβ and tau proteotoxicity have been developed to study their neurotoxic effects *in vitro* and *in vivo*, and have demonstrated that Aβ and tau together have worse cognitive and neurodegenerative consequences than either pathology on its own (Busche and Hyman, 2020)*.* Recent work characterizing additional co-pathologies in AD have identified a third protein, TDP-43, as a likely relevant contributor to AD pathophysiology (Josephs et al., 2015, 2014a; Kapasi et al., 2020; James et al., 2016).

TDP-43 was first identified in 2006 as the major component of pathologic inclusions in amyotrophic lateral sclerosis (ALS) and ∼50% of cases of frontotemporal lobar degeneration (FTLD-TDP) (Arai et al., 2006; Neumann et al., 2006). TDP-43 is an essential protein involved in multiple cellular processes, including alternative splicing of the majority of mRNA gene products, stabilization and transport of RNA transcripts, and the formation and stabilization of stress granules (Buratti and Baralle, 2008; Ratti and Buratti, 2016). Like tau, TDP-43 is subjected to post-translational modifications, including phosphorylation and acetylation, that promote aggregation (François-Moutal et al., 2019). Although the exact mechanisms continue to be studied, cytoplasmic mis-localization, phosphorylation and aggregation of TDP-43 contribute to neuronal dysfunction and neurodegeneration likely through both loss- and gain-of-TDP-43 functions (Kabashi et al., 2009; Gao et al., 2018). Most cases of ALS and FTLD-TDP are sporadic, without a known genetic cause. However, rare mutations in the gene encoding TDP-43, *TARDBP*, are causative for ALS, demonstrating that dysfunctional TDP-43 actively contributes to neurodegenerative pathways (Gendron et al., 2013; Van Deerlin et al., 2008).

Following its identification as a key pathologic protein in ALS and FTLD-TDP, TDP-43 pathology was also found to occur as a secondary pathology in other neurodegenerative diseases (Wang et al., 2008; Arai et al., 2009; Amador-Ortiz et al., 2007). In particular, TDP-43 co-pathology has been recognized in more than half of neuropathologically confirmed cases of AD (Josephs et al., 2014a,b, 2008, 2020; Jung et al., 2014; Nag et al., 2018; Keage et al., 2014; Uryu et al., 2008; Kadokura et al., 2009), and several large cohort studies have found that TDP-43 co-pathology is associated with significantly accelerated clinical progression in AD patients (Josephs et al., 2014a; Latimer et al., 2019; Chang et al., 2016), including more rapid rates of cognitive decline and increased mesial temporal atrophy (Josephs et al., 2017; Kapasi et al., 2020; James et al., 2016). Notably, concomitant TDP-43 pathology in AD occurs more frequently as tau pathology progresses into additional brain regions, denoted by higher Braak stage. The reported association between phosphorylated TDP-43 (pTDP-43) in hippocampus and faster hippocampal atrophy on MRI was limited to cases with higher Braak stages (III-VI) (Josephs et al., 2017), suggesting tau and TDP-43 may influence each other to promote neuronal dysfunction and neurodegeneration (James et al., 2016; Josephs et al., 2014b). In AD, neurofibrillary tangles and TDP-43-positive inclusions can co-exist or co-localize within a subset of neurons (Kadokura et al., 2009; Higashi et al., 2007; Smith et al., 2018), with ∼25% of phosphorylated TDP-43 immunopositive neurons also exhibiting pathological tau (PHF-1) immunoreactivity (Smith et al., 2018).

To date, there are limited studies exploring the relationship between tau and TDP-43 *in vitro* or *in vivo*. In cell and mouse models, TDP-43 regulates mRNA splicing of tau exon 10, shifting the ratio of tau microtubule binding repeats from the normal balanced ratio of 3R/4R-tau to a higher proportion of 4R-tau (Gu et al., 2017a). Recent work demonstrated that tau oligomers promote accumulation of cytoplasmic TDP-43 in HEK293 cells, and brain-derived TDP-43 oligomers can cross-seed tau aggregates *in vitro* (Montalbano et al., 2020). In *Caenorhabditis elegans*, we have shown that pan-neuronal co-expression of human tau and TDP-43 causes significant lethality, dramatically enhanced uncoordination, and an increase in accumulation of both total and phosphorylated tau and TDP-43 compared with controls expressing tau or TDP-43 alone (Latimer et al., 2019). However, the strong synthetic lethal phenotypes in this overexpression model preclude its use studying underlying causative relationships.

In order to dissect TDP-43 synergism with other pathological proteins, we have developed new refined models of co-morbid TDP-43 with the AD proteins tau and Aβ in *C. elegans*. Using these models, we show that very low levels of TDP-43 promote tau neurotoxicity, leading to significant sensory and behavioral impairments, accumulation of pathological phosphorylated tau, and selective neurodegeneration of neurons expressing specific neurotransmitter types. We further show that synergism between tau and TDP-43 is specific and does not extend to Aβ or poly-glutamine combined with TDP-43. Finally, we demonstrate that genetic loss-of-function of the tau modifier, *sut-2*, can robustly prevent the enhanced neurotoxicity and neurodegeneration observed in this model.

## RESULTS

To dissect the contribution of TDP-43 to exacerbated AD pathology-related neurotoxicity and neurodegeneration, we generated a new *C. elegans* strain with modest pan-neuronal expression of human TDP-43 (TDP Tg-low). Expression of TDP-43 in this strain is maintained at very low levels by the insertion of a self-cleaving ribozyme between the TDP-43 coding sequence and the 3′ untranslated region (UTR) of the transgene, which results in the inactivation of most, but not all, transgene-derived transcripts (Fig. S1) (Wurmthaler et al., 2019). In order to test whether TDP-43 potentiates neurotoxicity of the AD proteins tau or Aβ, TDP Tg-low animals were crossed with strains expressing wild-type human tau or Aβ1-42 pan-neuronally (tau Tg or Aβ Tg, respectively) to generate the co-expression strains tau+TDP Tg-low and Aβ+TDP Tg-low.

### TDP-43 synergizes with tau, but not Aβ, to worsen motility deficits

**Fig. 1.**
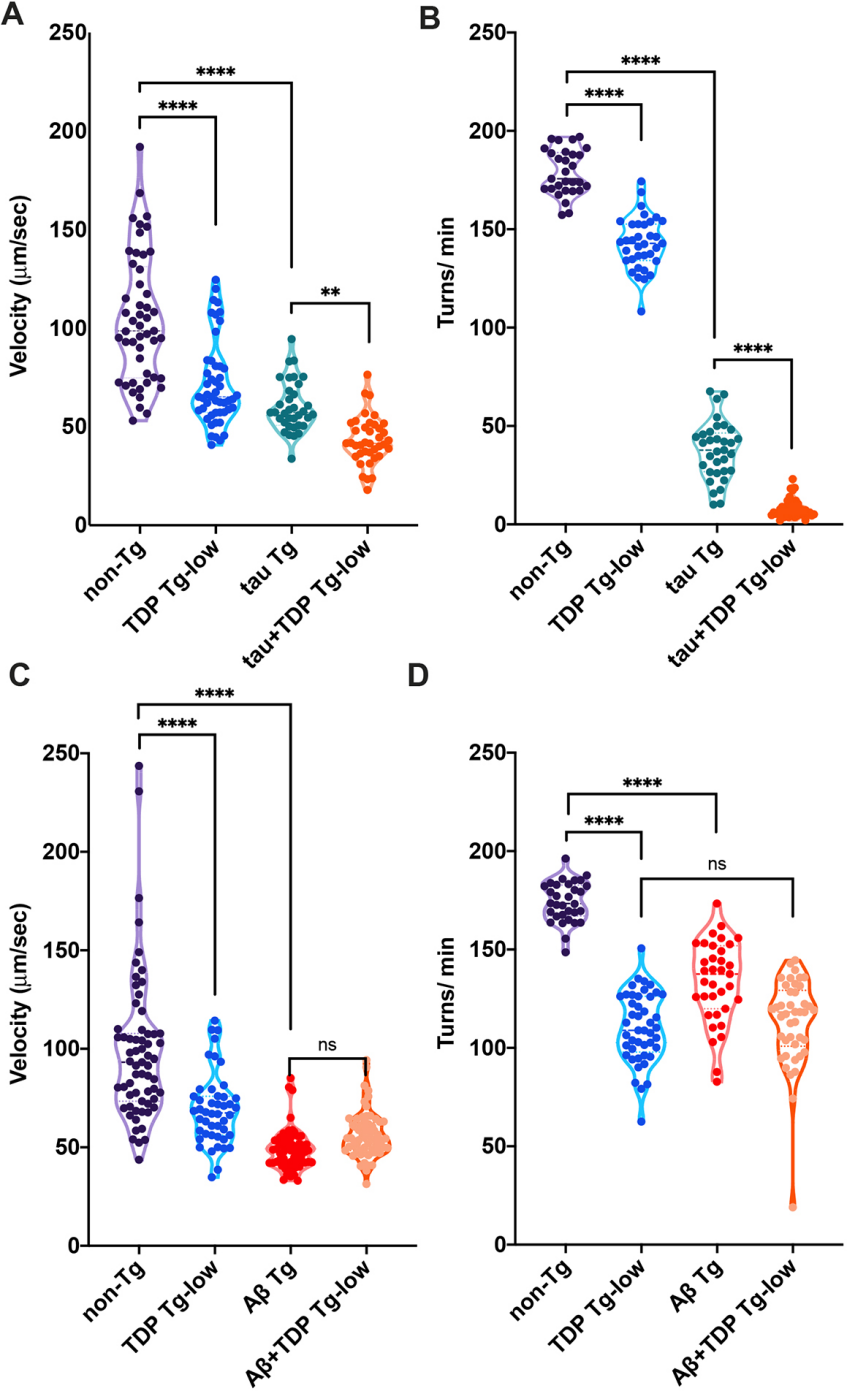
**Co-expression of TDP-43 with tau but not Aβ leads to enhancement of motility deficits*.*** (A) tau+TDP Tg-low animals have significantly decreased activity. Unstimulated activity on a seeded agar plate was detected using unbiased computer-assisted video tracking and analysis. Movement (velocity) was recorded (µm/sec). *n*=115 (non-TG), 66 (TDP Tg-low), 89 (tau Tg), 93 (tau+TDP Tg-low), from three independent replicates. (B) tau+TDP Tg-low animals showed significantly decreased thrashing in liquid. Rates of thrashing were measured using unbiased computer-assisted tracking and analysis. The number of turns (thrashes) per minute were recorded (turns/min). *n*=73 (non-TG), 89 (TDP Tg-low), 81 (tau Tg), 87 (tau+TDP Tg-low), from three independent replicates. (C) Unstimulated activity on a seeded agar plate was detected using unbiased computer-assisted video tracking and analysis. Movement (velocity) was recorded (µm/sec). *n*=147 (non-TG), 119 (TDP Tg-low), 163 (Aβ Tg), 182 (Aβ+TDP Tg-low), from three independent replicates. (D) Rates of thrashing in liquid were measured using unbiased computer-assisted tracking and analysis. The number of turns (thrashes) per minute were recorded (turns/min). *n*=85 (non-TG), 105 (TDP Tg-low), 101 (Aβ Tg), 119 (Aβ+TDP Tg-low), from three independent replicates. One-way ANOVA with Tukey's multiple-comparison test. **P<0.01, ****P<0.0001. ns, not significant.

Visually, the tau+TDP Tg-low animals exhibit dramatically worse motility than either tau Tg or TDP Tg-low strains, manifested by increased uncoordination in their movement. In fact, impaired motility has been used successfully as a readout for neuronal dysfunction or neurodegeneration in other *C. elegans* models of neurodegenerative disease (Kraemer et al., 2003; Wolozin et al., 2011) and represents the reciprocal functionality of both GABA-ergic and cholinergic neurons in motor circuits. To measure the severity of tau+TDP Tg-low uncoordination, we used automated video tracking to assay two different movement paradigms: unstimulated activity on a solid surface and thrashing in liquid. We found that these animals exhibited significantly decreased activity and worsened thrashing motility (Fig. 1A,B; Movies 1-8). In contrast, Aβ+TDP Tg-low animals exhibited no change in motility from control strains (Fig. 1C,D). To confirm these results, we generated an additional strain co-expressing a different Aβ transgene with TDP Tg-low (Aβ2+TDP Tg-low; Fong et al., 2016). Similarly, these animals exhibited no change in motility (Fig. S2).

**Fig. 2.**
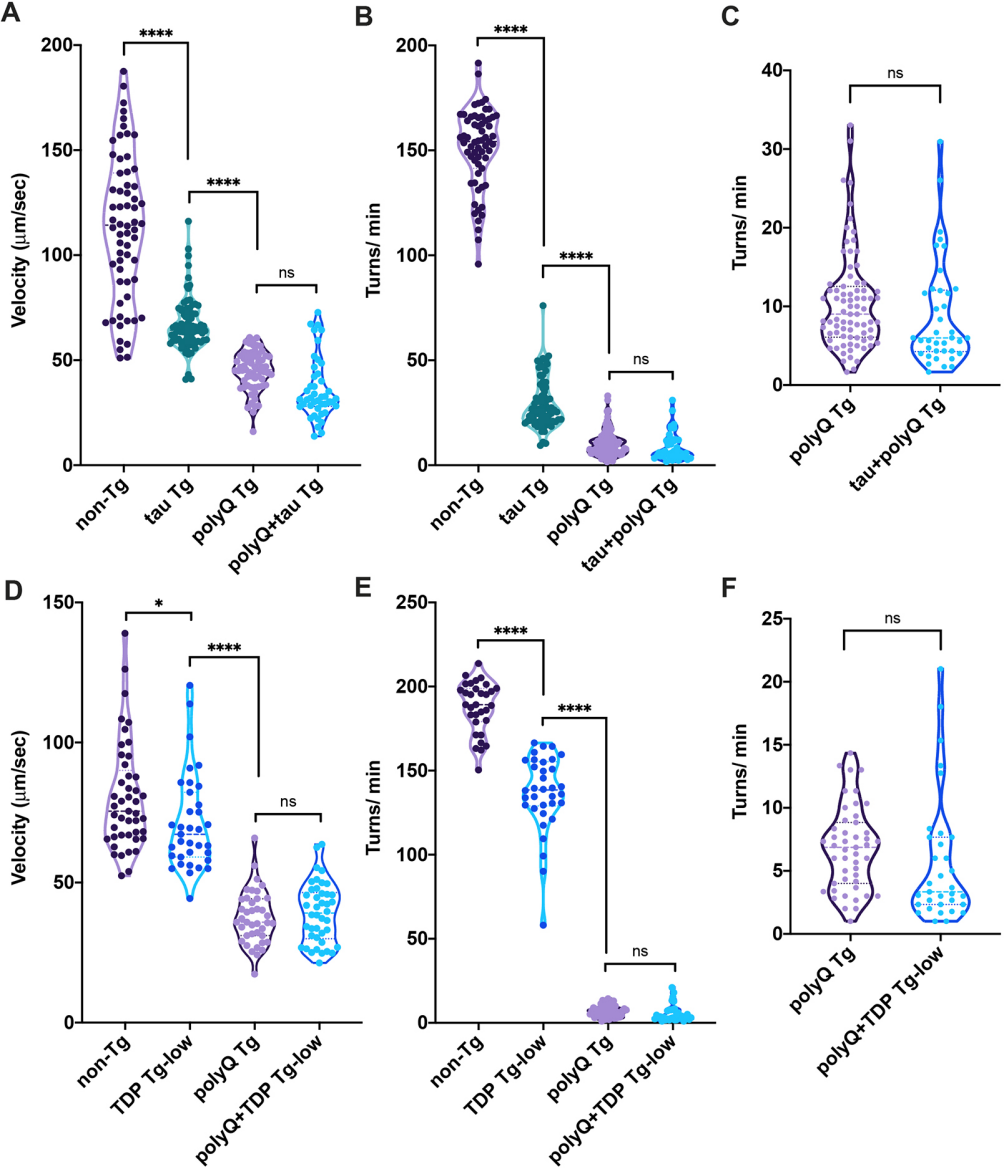
**tau and TDP-43 do not synergize with poly-glutamine.** (A-C) tau does not synergize with poly-glutamine. (A) Unstimulated activity on a seeded agar plate is detected using unbiased computer-assisted video tracking and analysis. Movement (velocity) was recorded (µm/sec). *n*=140 (non-TG), 179 (tau Tg), 138 (polyQ Tg), 110 (polyQ+tau Tg), from three independent replicates. (B) Rates of thrashing in liquid were measured using unbiased computer-assisted tracking and analysis. The number of turns (thrashes) per minute were recorded (turns/min). *n*=149 (non-TG), 204 (tau Tg), 167 (polyQ Tg), 132 (polyQ+tau Tg), from three independent replicates. (C) Data from B with decreased *y*-axis to allow visual comparisons between polyQ Tg and tau+polyQ Tg strains. (D-F) TDP-43 does not synergize with poly-glutamine. (D) Unstimulated activity on a seeded agar plate is detected using unbiased computer-assisted video tracking and analysis. Movement (velocity) was recorded (µm/sec). *n*=123 (non-TG), 99 (TDP Tg-low), 116 (polyQ Tg), 87 (polyQ+TDP Tg-low), from three independent replicates. (E) Rates of thrashing in liquid were measured using unbiased computer-assisted tracking and analysis. The number of turns (thrashes) per minute were recorded (turns/min). *n*=82 (non-TG), 94 (TDP Tg-low), 116 (polyQ Tg), 83 (polyQ+TDP Tg-low), from three independent replicates. (F) Data from E with decreased *y*-axis to allow visual comparisons between polyQ Tg and TDP+polyQ Tg strains. One-way ANOVA with Tukey's multiple-comparison test. *P<0.05, ****P<0.0001. ns, not significant.

As controls, we examined whether tau Tg or TDP Tg-low would synergize with a non-AD related neurodegenerative disease model expressing 86 repeats of polyglutamine pan-neuronally (polyQ Tg) (Brignull et al., 2006; Guthrie et al., 2009). PolyQ Tg animals accumulate insoluble inclusions of polyglutamine and have significant motility dysfunction on their own (Fig. 2). We have previously been able to distinguish enhancement of motility dysfunction from already impaired strains using assays detecting activity on a solid surface or thrashing in liquid (Liachko et al., 2016; Kraemer et al., 2006). In this case, we found no alteration in motility in either polyQ+tau Tg or polyQ+TDP Tg-low strains compared with controls (Fig. 2). Taken together, these experiments suggest that the neurotoxic synergy observed between tau and TDP-43 is specific rather than a non-specific enhancement of toxicity due to an increase in neuronal protein load, protein aggregation or merely additive toxicity through two independent pathological mechanisms.

### tau+TDP Tg-low animals exhibit impaired mechanosensation

**Fig. 3.**
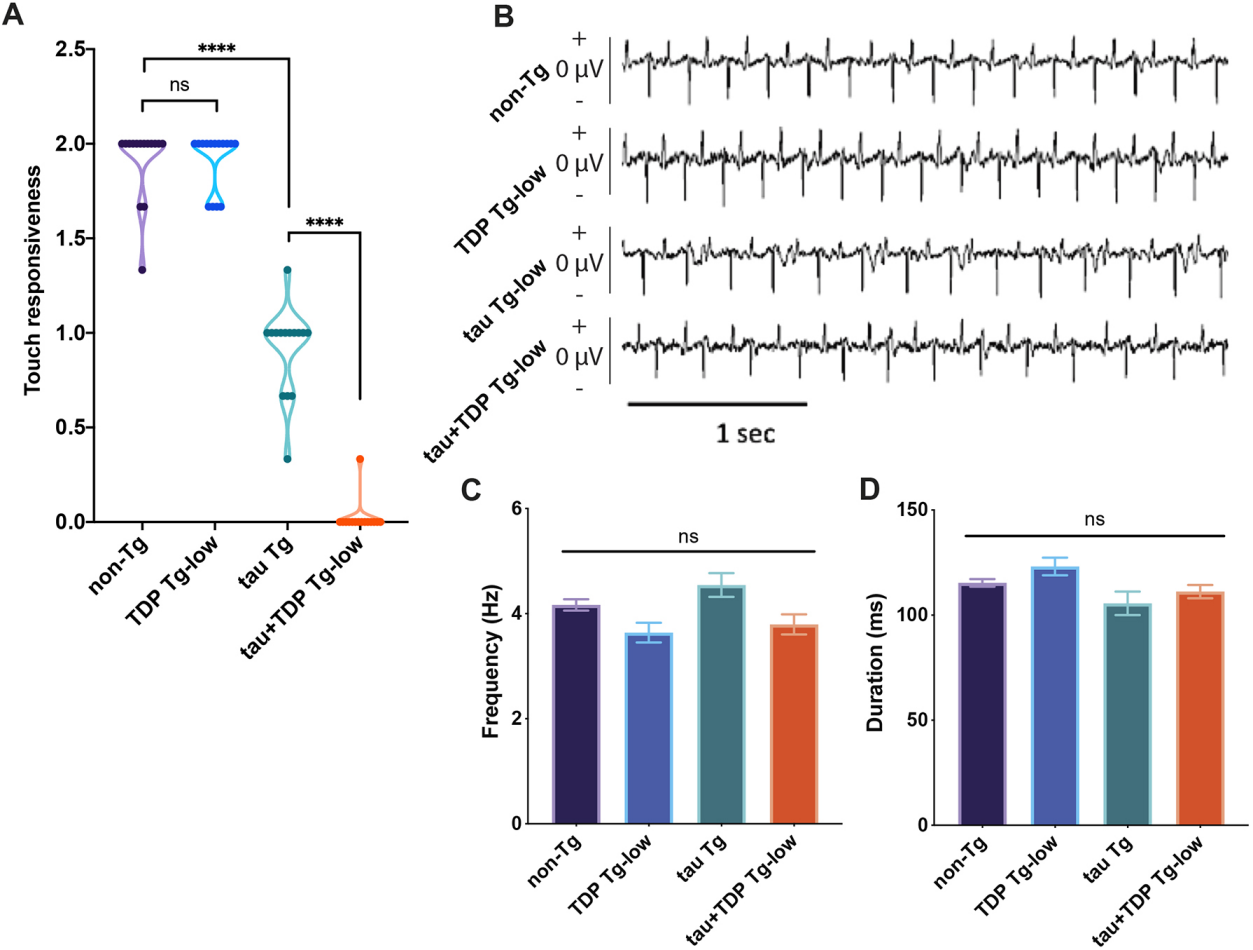
**tau+TDP Tg-low exhibit mechanosensory defects but intact pharyngeal pumping.** (A) tau+TDP Tg-low animals have significantly worse mechanosensation. Mechanosensation was assayed by responsiveness to a light touch and scored as: 2, normal response; 1, abnormal response; 0, no response. *n*=45 (all strains), from three independent replicates. (B-D) *C. elegans* pharyngeal pumping was evaluated by recording pharyngeal muscle and neuron action potentials to generate an electropharyngeogram (EPG). No significant differences were detected among strains. (B) Representative EPG traces of pharyngeal action potentials showing both positive (excitatory) and negative (relaxation) spikes. (C) Average pump frequency and (D) average pump duration over a 2 min recording. *n*=39 (non-TG), 36 (TDP Tg-low), 32 (tau Tg), 35 (tau+TDP Tg-low), from three independent replicates. One-way ANOVA with Tukey's multiple-comparison test. ****P<0.0001. ns, not significant. Data are mean±s.e.m.

We next examined several well-characterized behavioral outputs in the tau+TDP Tg-low model. Mechanosensation integrates signaling from dopaminergic, glutamatergic and serotonergic neurotransmitter classes of neurons to generate a stereotypical response to light touch. To evaluate this, we employed an assay evaluating responses to gentle touch with an eyelash hair ten times, alternating head and tail touches. Consistent with previous reports (Miyasaka et al., 2005; Guha et al., 2020; Pir et al., 2017), tau Tg animals had a moderate defect in mechanosensation (Fig. 3A). Interestingly, we found that tau+TDP Tg-low animals had significantly worsened defects in mechanosensation. We also tested whether tau+TDP Tg-low animals had altered pharyngeal pumping (feeding behavior), an activity that integrates signaling from serotonergic, octopaminergic and cholinergic neurotransmitter classes of neurons. To assay this, we employed microfluidic electropharyngeogram (EPG) recording to measure electrical currents emitted by pharyngeal muscles and neurons (Liu et al., 2019; Lockery et al., 2012). However, we did not observe any significant effects on the frequency or duration of pharyngeal pumping in tau+TDP Tg-low (Fig. 3B-D), indicating that the circuits controlling this behavior remain intact.

### TDP-43 promotes increased accumulation of total and phosphorylated tau

**Fig. 4.**
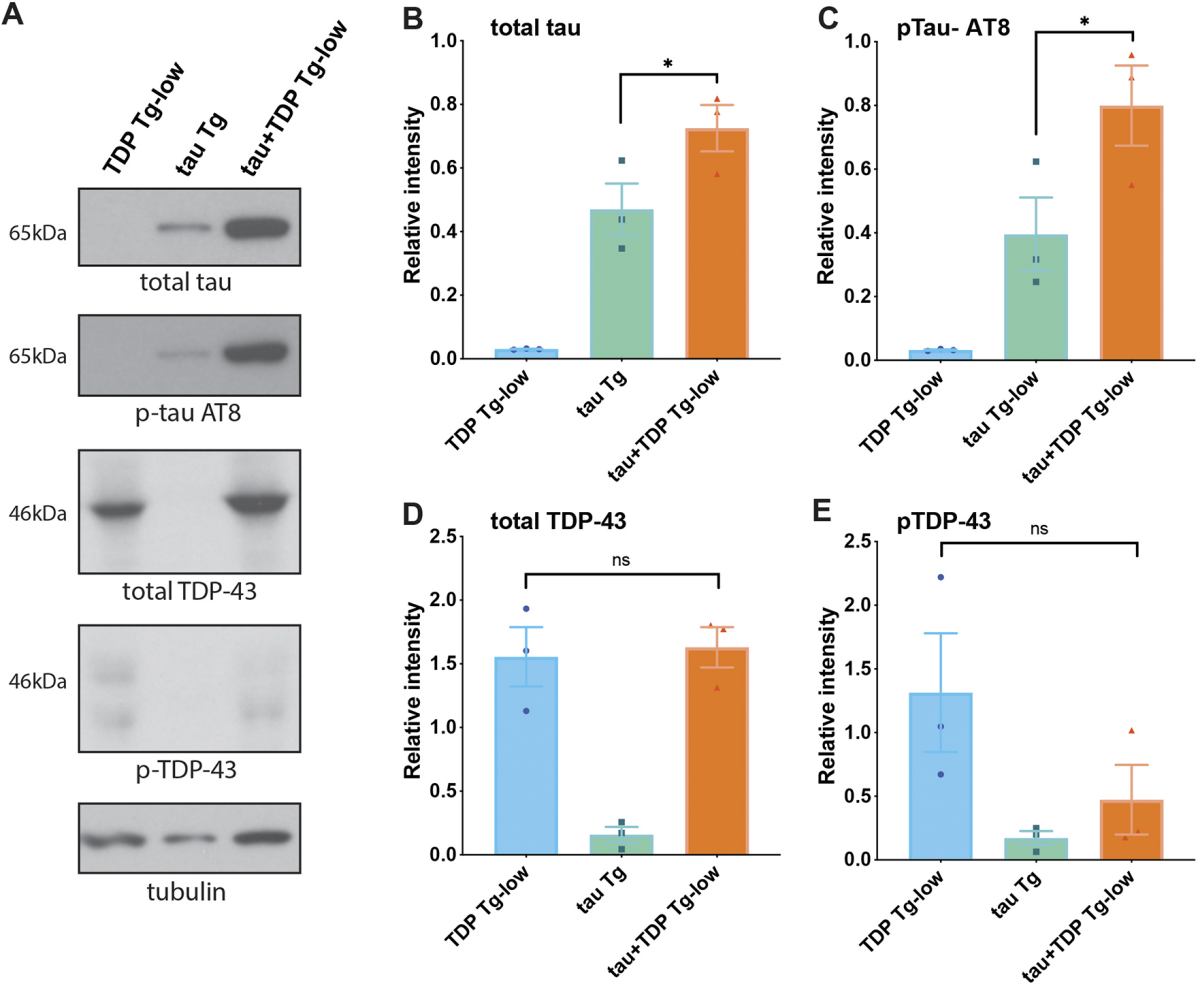
**tau and TDP-43 co-expression promotes accumulation and phosphorylation of tau.** (A-E) Developmentally synchronized day 1 adult *C. elegans* were harvested and tested by immunoblot for total tau, phosphorylated tau (AT8), total TDP-43, phosphorylated TDP-43 (phospho-S409/410) and tubulin (load control). (A) Immunoblot shown is representative of three independent replicate experiments. (B-E) Quantification of protein levels normalized to tubulin. Total tau (B) and phosphorylated tau (C) are elevated in tau+TDP-low Tg animals. Total TDP-43 (D) and phosphorylated TDP-43 (E) are not elevated in tau+TDP-low Tg animals. One-way ANOVA with Tukey's multiple-comparison test. *P<0.05. ns, not significant. Data are mean±s.e.m.

It is possible that the motility and sensory impairments observed in tau+TDP Tg-low are due to accumulation of neurotoxic pathological tau or TDP-43. To determine whether the co-expression of tau and very low levels of TDP-43 influenced their protein accumulation, we measured protein levels in tau+TDP Tg-low by immunoblotting. We found that both total and phosphorylated tau increased in tau+TDP Tg-low (Fig. 4A-C), but, surprisingly, protein accumulation of TDP-43 was unchanged from TDP-43 Tg-low alone and there was no apparent accumulation of phosphorylated TDP-43 (Fig. 4A,D,E). We tested whether differences in tau protein levels in tau+TDP Tg-low animals were due to increased mRNA expression, but we did not see any significant differences from tau Tg alone (Fig. S3). Therefore, TDP-43 potentiates pathological tau protein changes, which may be the primary driver of neurotoxicity in co-expression models of tau and TDP-43.

### tau+TDP Tg-low animals exhibit selective neuronal vulnerability through aging

**Fig. 5.**
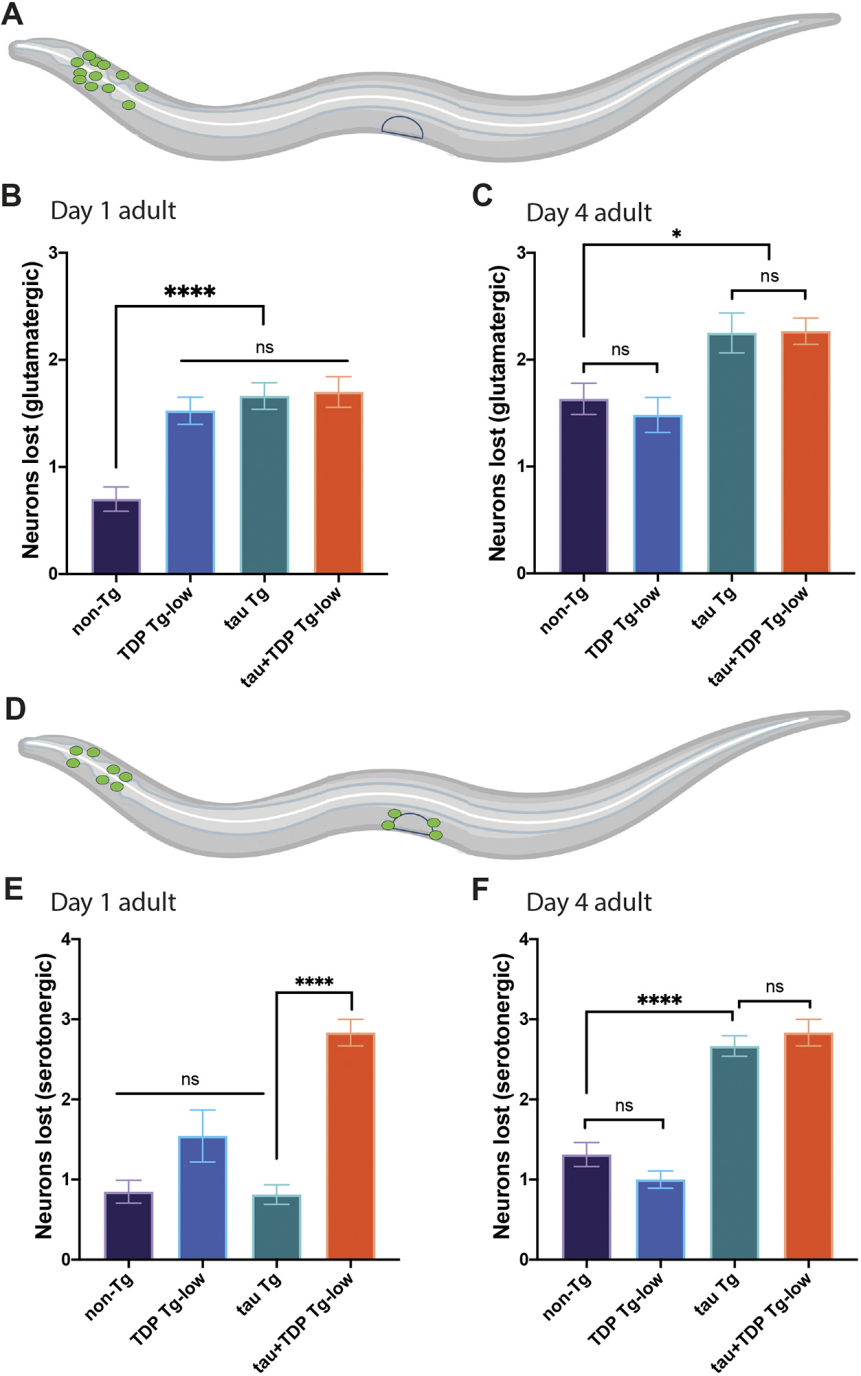
**Co-expression of tau and TDP-43 leads to selective glutamatergic and serotonergic neurodegeneration.** (A-C) Assessment of glutamatergic neurons in tau+TDP Tg-low animals. Depiction of neurons scored (green) (A). Quantification of neurons lost in developmentally synchronized day 1 adult (B) and day 4 adult (C) *C. elegans. n*>40 for all strains and time points scored, from at least three independent replicates. (D-F) Assessment of serotonergic neurons in tau+TDP Tg-low animals. Depiction of neurons scored (green) (D). Quantification of neurons lost in developmentally synchronized day 1 adult (E) and day 4 adult (F) *C. elegans. n*>45 for all strains and time points scored, from at least three independent replicates. One-way ANOVA with Tukey's multiple-comparison test. *P<0.05, ****P<0.0001. ns, not significant. Data are mean±s.e.m.

**Fig. 6.**
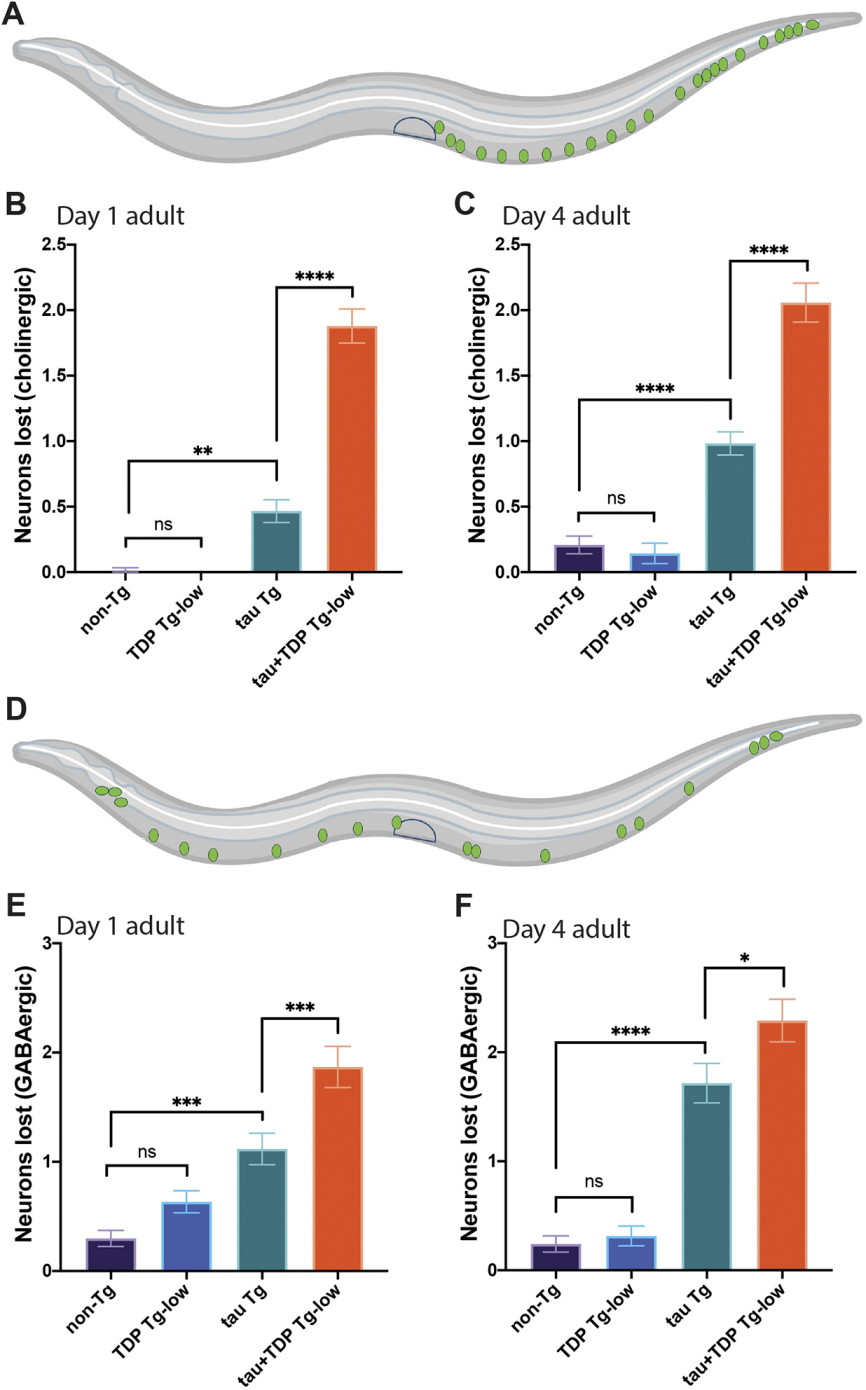
**Co-expression of tau and TDP-43 leads to selective cholinergic and GABA-ergic neurodegeneration.** (A-C) Assessment of cholinergic neurons in tau+TDP Tg-low animals. Depiction of neurons scored (green) (A). Quantification of neurons lost in developmentally synchronized day 1 adult (B) and day 4 adult (C) *C. elegans. n*>45 for all strains and time points scored, from at least three independent replicates. (D-F) Assessment of GABA-ergic neurons in tau+TDP Tg-low animals. Depiction of neurons scored (green) (D). Quantification of neurons lost in developmentally synchronized day 1 adult (E) and day 4 adult (F) *C. elegans. n*>45 for all strains and time points scored, from at least three independent replicates. One-way ANOVA with Tukey's multiple-comparison test. *P<0.05, **P<0.01, ***P<0.001, ****P<0.0001. ns, not significant. Data are mean±s.e.m.

To determine whether the motor and sensory defects of tau+TDP Tg-low are the result of neurodegeneration, we assayed for neuronal integrity in these animals using GFP reporters specific for distinct neurotransmitter classes of neurons (dopaminergic, glutamatergic, serotonergic, cholinergic or GABA-ergic). We also evaluated whether aging differentially affected these various neurotransmitter classes of neurons by assessing neurodegeneration over time at day 1 and day 4 of adulthood. This survey revealed distinct differences in neuron vulnerabilities by neurotransmitter class to co-morbid tau and TDP-43, and to aging. We found no significant difference in dopaminergic neurons at either day 1 or day 4 of adulthood between all strains (Fig. S4). Conversely, all transgenic strains surveyed had a significant decrease in glutamatergic neurons compared with non-Tg animals at day 1 of adulthood; however, by day 4 of adulthood, both tau Tg and tau+TDP Tg-low animals had lost significantly more neurons than non-Tg or TDP Tg-low animals alone (Fig. 5A-C). Interestingly, we saw a different pattern of loss in serotonergic neurons, where at day 1 of adulthood only tau+TDP Tg-low animals lost significantly more neurons than controls (Fig. 5D,E). By day 4 of adulthood, tau Tg animals exhibited significant neurodegeneration; however, at this age, neuron loss in tau+TDP Tg-low animals did not progress further and was not significantly different from tau Tg animals (Fig. 5F). In cholinergic and GABA-ergic type neurons still another pattern of neurodegeneration was noted. Although both tau Tg and tau+TDP Tg-low Tg animals exhibited significant cholinergic and GABA-ergic neuronal loss compared with controls at day 1 and day 4 of adulthood, the neuron loss was most severe in tau+TDP Tg-low animals at both time points (Fig. 6).

### Loss of *sut-2* prevents protein accumulation and motility deficits in tau+TDP Tg-low animals

**Fig. 7.**
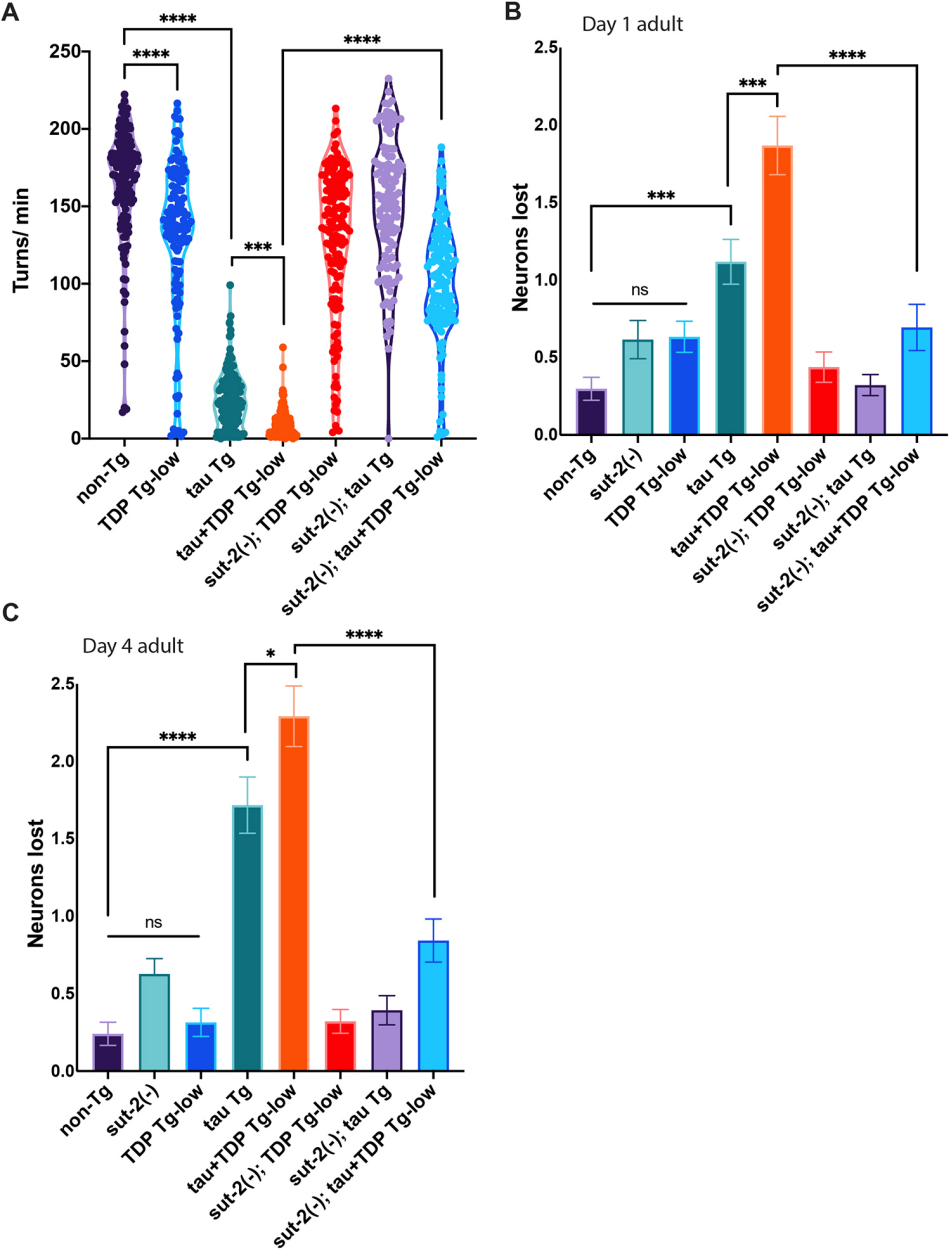
**Loss of *sut-2* expression prevents motility deficits and neurodegeneration in a model of combined tau and TDP-43 expression.** (A) tau+TDP Tg-low animals have significantly decreased thrashing in liquid, which is rescued by *sut-2(−)*. Rates of thrashing were measured using unbiased computer-assisted tracking and analysis. The number of turns (thrashes) per minute were recorded (turns/min). ***P<0.0008, ****P<0.0001. TDP-43 Tg-low versus *sut-2(−);* TDP Tg-low: *P*=0.9833; tau Tg versus *sut-2(−);* tau Tg: *P*<0.0001; tau+TDP Tg-low versus *sut-2(−);* tau+TDP Tg-low: *P*<0.0001. *n*=149 (non-TG), 134 (TDP Tg-low), 167 (tau Tg), 185 (tau+TDP Tg-low), 145 (*sut-2(−);* TDP Tg-low), 121 (*sut-2(−)*; tau Tg), 131 (*sut-2(−);* tau+TDP Tg-low) from three independent replicates. (B,C) Quantification of neurons lost in developmentally synchronized day 1 adult (B) and day 4 adult (C) *C. elegans* show that *sut-2(−)* prevents neuron loss in tau+TDP Tg-low animals*.* *P<0.05; ***P<0.001; ****P<0.0001. (B) TDP-43 Tg-low versus *sut-2(−);* TDP Tg-low: *P*=0.9504; tau Tg versus *sut-2(−)*; tau Tg: *P*=0.0002; tau+TDP Tg-low versus *sut-2(−);* tau+TDP Tg-low: *P*<0.0001. (C) TDP-43 Tg-low versus *sut-2(−);* TDP Tg-low: *P*>0.9999; tau Tg versus *sut-2(−);* tau Tg: *P*<0.0001; tau+TDP Tg-low versus *sut-2(−);* tau+TDP Tg-low: *P*<0.0001. *n*>45 for all strains and time points scored, from at least three independent replicates. One-way ANOVA with Tukey's multiple-comparison test. ns, not significant. Data are mean±s.e.m.

**Fig. 8.**
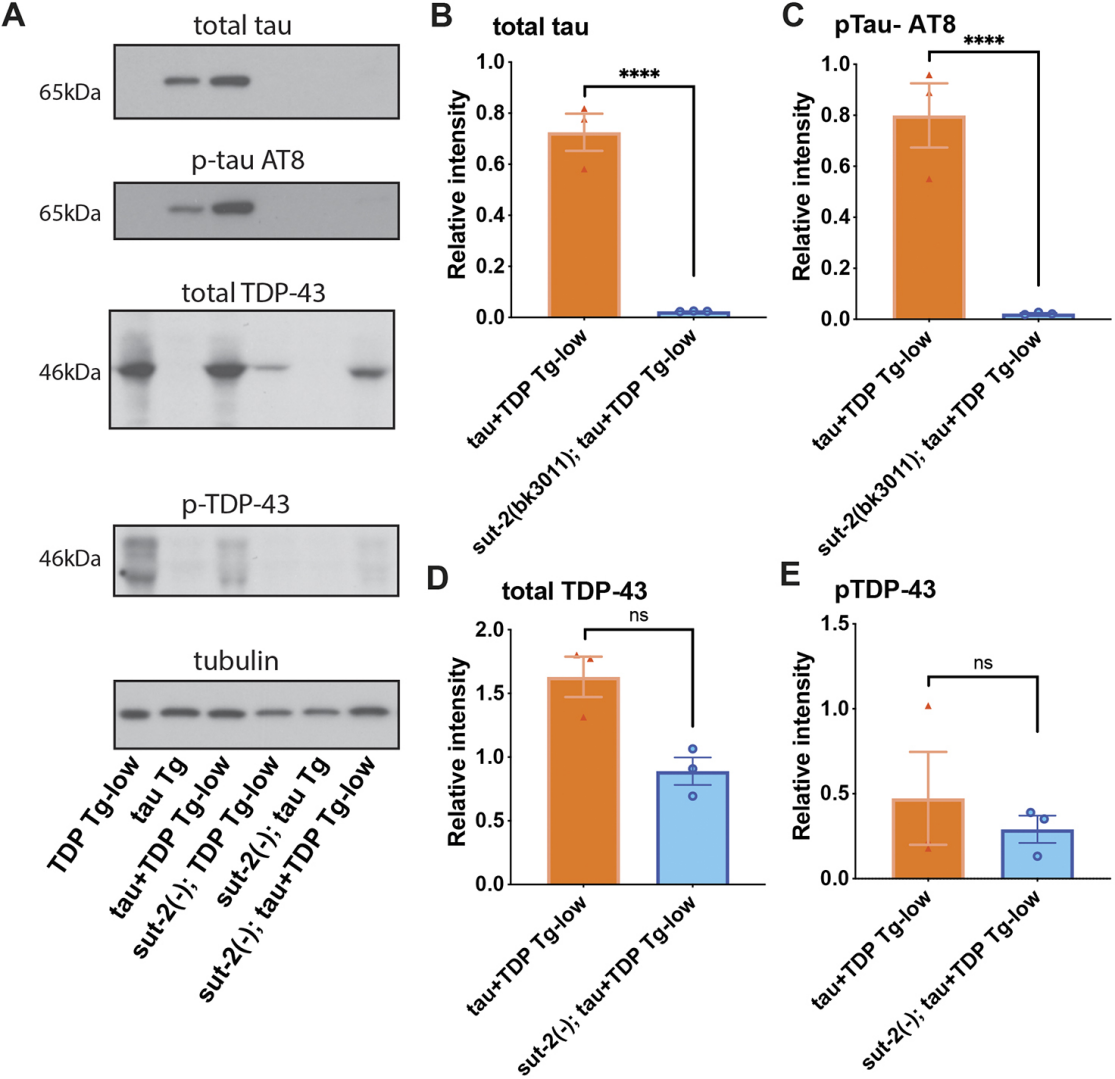
**Loss of *sut-2* expression prevents tau protein accumulation in a model of combined tau and TDP-43 expression.** (A-E) Developmentally synchronized day 1 adult *C. elegans* were harvested and tested by immunoblot for total tau, phosphorylated tau (AT8), total TDP-43, phosphorylated TDP-43 (phospho-S409/410) and tubulin (load control). Immunoblot shown (A) is representative of three independent replicate experiments. (B-E) Quantification of protein levels performed by ImageJ software analysis of scanned film images. Total tau (B) and phosphorylated tau (C) are reduced in *sut-2(−);* tau+TDP-low Tg animals. Total TDP-43 (*P*=0.0917) (D) and phosphorylated TDP-43 (E) are not significantly reduced in *sut-2(−);* tau+TDP-low Tg animals. One-way ANOVA with Tukey's multiple-comparison test. ****P<0.0001. ns, not significant. Data are mean±s.e.m.

To test whether suppression of tau toxicity can protect against the phenotypes of co-expressed tau and TDP-43, we used a CRISPR-Cas9-generated whole-gene deletion mutation in a known, well-characterized suppressor of tau, *sut-2/MSUT2* (also known as *ZC3H14*) (Guthrie et al., 2009, 2011; Wheeler et al., 2010)*.* We found *sut-2* null mutations [*sut-2 (−)*] robustly protected against tau+TDP Tg-low uncoordinated motility (Fig. 7A) and GABA-ergic neurodegeneration (Fig. 7B,C). We then examined whether *sut-2(−)* could reduce the accumulation of tau or TDP-43 protein. We found that *sut-2(−)* reduced both total tau protein and pathological phosphorylated tau in tau+TDP Tg-low animals (Fig. 8A-C). However, *sut-2(−)* did not significantly reduce levels of total or phosphorylated TDP-43 in tau+TDP Tg-low (Fig. 8A,D,E)*.* Taken together, these data suggest that enhanced tau neurotoxicity underlies the synergism observed between tau and TDP-43.

## DISCUSSION

Through a series of functional experiments in a *C. elegans* model of combined tau and TDP-43 proteotoxicity, we demonstrate that the presence of TDP-43 promotes the accumulation and hyperphosphorylation of tau, whereas tau does not have the same effect on TDP-43 protein. We also show that the combination of tau and TDP-43 differentially affects neurons of different neurotransmitter classes, indicating some degree of selective cell-type vulnerability. Further, we show that suppression of tau neurotoxicity using the genetic modifier *sut-2* protects against effects of tau and TDP-43 synergism.

### TDP-43 promotes tau-driven neurotoxicity and neurodegeneration

The attenuated model of co-expressed tau and TDP-43 described here uses a TDP-43 transgene with very low pan-neuronal expression of human TDP-43, and accumulates minimal measurable phosphorylated TDP-43. Neither total nor phosphorylated TDP-43 protein levels significantly change in the combined tau+TDP-43-low Tg animals, although there may be biologically relevant differences that did not reach statistical significance in these experiments. This suggests that tau does not promote pathological TDP-43 protein changes as robustly as TDP-43 enhances tau pathology and, therefore, the worsened behavioral phenotypes and neurodegeneration are less dependent on increased total or phosphorylated TDP-43. Conversely, the observed significant increase in both total and phosphorylated tau in the tau+TDP-43-low Tg animals indicates that low levels of non-phosphorylated wild-type TDP-43 can drive increased tau pathology with concomitant worsened neuronal function and neurodegeneration. We tested whether loss of the endogenous *C. elegans* TDP-43 homolog, *tdp-1*, impacted neurotoxicity of tau Tg animals, but did not see any difference in tau-mediated motility dysfunction (Fig. S5). The *C. elegans tdp-1* gene lacks the C-terminal G-rich domain present in human TDP-43, which is the site of pathological phosphorylation, the location of most ALS-causing mutations in the *TARDBP* gene, and comprises the fibrillar core of aggregates in FTLD-TDP (De Boer et al., 2020; Arseni et al., 2021). It is possible that this C-terminal domain is a crucial region involved in synergy with tau*.* The relationship between tau and TDP-43 is likely complex, and although these results do not exclude a reciprocal effect of tau on TDP-43, they do support TDP-43 promoting tau-driven neurotoxicity and neurodegeneration as a major component of the underlying mechanism.

Previous studies evaluating a potential relationship between tau and TDP-43 have suggested that tau initiates TDP-43 pathology (Clippinger et al., 2013). Although this relationship is not observed in tau+TDP Tg-low animals, our previous, more severe, model employing higher co-expression of tau and TDP-43 did show a strong increase in both total and phosphorylated tau and TDP-43 protein (Latimer et al., 2019). These differences may be due to a threshold effect, below which tau does not impact TDP-43, and above which it reciprocally enhances TDP-43 pathology. Additional work is necessary to further tease apart these complex pathological protein interactions.

### Neurotransmitter classes of neurons are selectively vulnerable to co-morbid tau and TDP-43

We find that different neurotransmitter classes of neurons are selectively vulnerable to co-expression of tau and TDP-43. Dopaminergic neurons were resistant to tau and TDP-43, whereas glutamatergic, serotonergic, cholinergic and GABA-ergic neurons all exhibited worsened neuronal loss. Interestingly, these neuron classes also exhibited differences through aging and among neurotransmitter class. In glutamatergic neurons, tau Tg and tau+TDP Tg-low animals had similar degrees of neurodegeneration over time, indicating that TDP-43 does not worsen tau-driven neuronal loss in these cells. In serotonergic neurons, TDP-43 appears to accelerate early tau-driven neurodegeneration, but not worsen the total neurodegeneration accumulating over time. Finally, co-expression of tau and TDP-43 in cholinergic and GABA-ergic neurons led to significantly increased neuronal loss at each time point surveyed. Although it is possible that differences in *snb-1* or *aex-3* expression among these neurotransmitter classes may underlie the observed vulnerabilities, we do not believe this is the case, as both *snb-1* and *aex-3* are robustly expressed across neurons, including dopaminergic neurons which were resistant to combined tau+TDP (Taylor et al., 2021). There have been considerable efforts made to understand the selective vulnerability of neurons to different pathologic processes in neurodegenerative diseases including AD (Saxena and Caroni, 2011). In terms of the various neurotransmitter classes, evidence suggests that all are to some degree affected in AD, but the underlying mechanisms driving their vulnerabilities and the temporal patterns of their involvement are poorly understood.

Dysfunction of both glutamatergic and cholinergic neurons in AD is well described (Francis, 2005; Cheng et al., 2021). Glutamate, the primary excitatory neurotransmitter in the human brain, has been shown to decrease both with age and AD pathology (Cowburn et al., 1988; Mcentee and Crook, 1993). Similarly, cholinergic synapses have long been implicated in the pathophysiology of AD given their prominence throughout the limbic system and neocortex, regions that coincide with both progressive tau and TDP-43 pathology (Etienne et al., 1986; Wevers and Schröder, 1999). In our model, the cholinergic neurons of animals with both tau and TDP-43 were more significantly affected than those with either tau or TDP-43 alone at both ages.

Although less well studied, GABA-ergic neurons have more recently been shown to play a role in AD. GABA-ergic neurons are involved in cortical microcircuits that are affected in AD, and increasing evidence supports AD deficits linked to GABA-ergic inhibitory neuron dysfunction (Mattson, 2020; Xu et al., 2020; Ruan et al., 2021). The serotonergic system is also implicated in AD (Trillo et al., 2013; Šimić et al., 2017) and some studies suggest that loss of serotonergic input is associated with early behavioral changes in AD, including increased agitation and loss of emotional regulation, clinical features that manifest before the later onset of memory loss and may also correlate with concomitant TDP-43 pathology (Sennik et al., 2017; Elferink et al., 2015; Simic et al., 2009). Although cell type vulnerability observed in our model is intriguing, further characterization of tau and TDP-43 pathology in these neuronal subtypes in human brain tissue will be necessary to better understand both the relevance of the model and the pathophysiology of the human disease.

### The synergistic relationship between tau and TDP-43 is specific

To determine the specificity of the neurotoxic synergy observed between tau and TDP-43, we tested whether TDP-43 could synergize with Aβ, another pathological AD protein. However, the Aβ+TDP Tg animals showed no enhancement of movement impairment compared with the lowest motility single transgenic strain (either TDP-43 or Aβ Tg animals). Notably, it has been previously shown that tau synergizes with Aβ in *C. elegans* using the same transgenes used here (tau Tg and Aβ Tg) (Benbow et al., 2020)*.* We also tested whether a neurotoxic non-AD neurodegenerative disease-associated protein, 86 repeats of poly-glutamine, could synergize with tau or TDP-43. However, we did not observe any enhancement of movement impairment in polyQ+tau Tg or polyQ+TDP Tg-low compared with polyQ Tg alone. Taken together, these data support the idea that tau and TDP-43 have a biologically relevant interaction and, therefore, the phenotypes observed in tau+TDP Tg animals are not simply due to neuronal protein load or non-specific additive toxicities of two distinct pathologic proteins.

Interestingly, in our models of co-expressed Aβ and TDP-43, we see no evidence of synergism between them. There is limited data on potential relationships between TDP-43 and Aβ reported in the literature, which includes some human autopsy studies. In a predictive model of neuropathological pathways, neuritic amyloid plaques had a significant effect on TDP-43 pathology (Power et al., 2018). However, neuritic plaques represent a combined lesion that includes both pathologic tau and Aβ, as well as more advanced disease. Data associating TDP-43 with diffuse Aβ pathology irrespective of tau is not reported and, indeed, a study examining TDP-43 pathological correlates with antemortem Aβ found no association with global amyloid PET signal (Teipel et al., 2021). In experimental systems, *in vitro* TDP-43 does not modulate the expression of APP, nor does APP affect TDP-43 expression (Hicks et al., 2020), but TDP-43 can accelerate Aβ aggregation in an *in vitro* seeding assay (Laos et al., 2021). In a separate study, injection of TDP-43 into brains of a transgenic mouse model of AD β-amyloidosis altered β-amyloid assembly and increased accumulation of toxic β-amyloid oligomers (Shih et al., 2020). Thus, the relationship between TDP-43 and Aβ needs further investigation.

### Loss of the potent tau modifier *sut-2* prevents tau and TDP-43 synergistic neurotoxicity

In studies evaluating the role of *sut-2* in cell culture, *C. elegans* and mouse models of tau neurotoxicity, genetic loss of *sut-2* protects against accumulation of insoluble tau, and prevents tau-mediated behavioral and cognitive decline and neurodegeneration (Guthrie et al., 2009, 2011; Kraemer and Schellenberg, 2007). Here, we find that loss of *sut-2* also suppresses the neurotoxicity of co-expressed tau and TDP-43. *sut-2(−)* only modestly decreases TDP-43 protein accumulation and does not protect against TDP Tg motility impairment. The near complete amelioration of motility defects and neurodegeneration in the combined tau+TDP-low Tg model in the presence of *sut-2(−)* supports the concept that enhanced tau neurotoxicity drives the exacerbated phenotypes. However, the mechanism of this protection remains unknown. SUT-2 is nuclear localized, binds mRNA and regulates poly-A tail length (Guthrie et al., 2009; Kow et al., 2021; Mcmillan et al., 2021; Grenier St-Sauveur et al., 2013). *sut-2* activity may drive mRNA into the cytoplasm, which can serve as a polyanion seed for tau aggregation. Loss of *sut-2* may decrease export of mRNA into the cytoplasm, reducing RNA-mediated tau aggregation and neurotoxicity (Kampers et al., 1996; Ginsberg et al., 1997).

### The underlying mechanisms by which TDP-43 promotes tau pathology are not understood

Human neuropathology data demonstrate that both tau and TDP-43 pathology occur in the same brain regions and can co-exist within the same neurons (Arai et al., 2009; Amador-Ortiz et al., 2007; Chornenkyy et al., 2019). Furthermore, phosphorylated tau and TDP-43 derived from an AD brain can co-immunoprecipitate, indicating a physical interaction (Tomé et al., 2021). Normal TDP-43 is predominantly localized to the nucleus and tau to the cytoplasm, making a direct interaction unlikely under homeostatic cellular conditions. However, TDP-43 can shuttle to the cytoplasm to carry out some of its physiologic functions, and redistribution of TDP-43 to the cytoplasm is believed to be an important part of the pathologic process. Classical pathological TDP-43 aggregates are found within the cytoplasm as perinuclear inclusions or other structures, in addition to intranuclear pathology. Further, although tau is best studied for its roles in microtubule assembly and stability, it can also be found in the nucleus where it is capable of binding DNA and may stabilize heterochromatin (Bukar Maina et al., 2016). Therefore, a direct interaction cannot be completely excluded; indeed, recent work provides compelling evidence for a direct interaction between pathological TDP-43 and pathological tau (Tomé et al., 2021), and previous ultrastructural studies have shown TDP-43 within tau structures in neurons, both in animal models and human tissues (Clippinger et al., 2013; Lin and Dickson, 2008). Determining whether individual domains of TDP-43 contribute to this synergy will be important future work.

Aside from a direct interaction, TDP-43 has multiple roles that could ultimately impact tau and tau pathology more indirectly, such as regulation of RNA splicing (White et al., 2018). TDP-43 directly binds to and regulates the splicing of most cellular pre-mRNAs, including tau mRNA, which has been shown to alter the ratio of tau isoforms implicated in neurodegenerative disease and may represent one mechanism of tau pathology exacerbation (Gu et al., 2017b). More extensive work in this model system and others are needed to further investigate the mechanism of TDP-43 enhancement of tau pathology.

### TDP-43 may act through additional pathways unrelated to tau pathology

In addition to enhancing tau pathology, TDP-43 can promote neurodegeneration through separate, tau-independent pathways. In human diseases, there are primary TDP-43 proteinopathies, such as ALS and FTLD-TDP, which are characterized by TDP-43 pathology in the absence of tau pathology. Mutations in the gene coding for TDP-43 cause some cases of ALS (Hortobágyi and Cairns, 2017) and numerous animal models have shown aberrant TDP-43 promotes neurodegeneration (Tan et al., 2017). Our model of combined tau and TDP-43 illustrates that, although TDP-43 is capable of causing neurodegenerative changes on its own, when tau is present it can additionally act to exacerbate tau pathology and set into motion a neurodegeneration cascade more severe than if either protein were present alone.

### tau and TDP-43 synergy represents a novel therapeutic target for treating AD

There is a great clinical need for effective AD therapeutics, as currently there are no treatments that arrest disease progression in AD. Co-morbid TDP-43 occurs in over half of patients with AD, and correlates with more severe disease course and greater neurodegeneration. We have shown that TDP-43 selectively enhances tau neurotoxicity, providing a possible mechanism for the clinical impact of TDP-43 in AD. Because the underlying mechanisms may be distinct, it is possible that the treatment for patients with both AD and TDP-43 pathology will need to be different from those for patients that lack this co-morbidity. Modulation of the tau suppressor *MSUT2* may be another viable target to intervene in AD with co-morbid TDP-43. A therapy that reduced or prevented TDP-43 and tau synergy could represent a novel and effective strategy to treat AD. To achieve this, continued development and testing of experimental models that incorporate multiple pathological proteins, and in particular TDP-43, is essential. These models will allow a deeper understanding of underlying mechanisms, as well as a system to test AD-targeting interventions.

### Conclusions

The role of pathological TDP-43 in AD is only just now becoming widely appreciated despite published neuropathologic descriptions dating back to 2006, and there are few published models of this combined pathology. Here, we demonstrate that modeling co-morbid tau and TDP-43 is relevant and necessary for advancing our understanding of the proteotoxic pathways underlying AD. We show that TDP-43 can exacerbate tau pathology, setting into motion a neurodegeneration cascade more severe than if either protein were present alone, and dependent on the neuron subtype. The robust amelioration of the combined phenotype by the loss-of-function of a known potent tau modifier, *sut-2*, further supports the notion that the enhanced phenotype is largely due to a TDP-43-driven exacerbation of tau pathology. Our extensive characterization of this *C. elegans* model of tau and TDP-43 co-expression provides a baseline upon which to build and improve our understanding of how these proteins interact to exacerbate neurodegenerative pathways. Synergy between tau and TDP-43 represents a novel therapeutic target for AD with TDP-43 pathology. Continued work using simple and translational models will be crucial for further probing those mechanisms and identifying treatment strategies.

## MATERIALS AND METHODS

### *C. elegans* strains and transgenics

Wild-type *C. elegans* (Bristol strain N2) was maintained as previously described (Brenner, 2003). Previously generated transgenic strains used were as follows: CK1441 *bkIs1441[Paex-3::Tau WT(4R1N)+Pmyo-2::dsRED]* (Benbow et al., 2020), GRU102 *gnaIs2[Pmyo-2::YFP+Punc-119::Abeta1-42]* (Fong et al., 2016), CL2355 *dvIs50[pCL45(snb-1::Abeta1-42::3′UTR(long))+mlt-2::GFP]* (Wu et al., 2006), CK241 *bkIs241[pF25B5.3::Q86-YFP]* (Guthrie et al., 2009) and EG1285 *oxIs12[Punc-47*::GFP *+ lin-15(+)*] (Mcintire et al., 1997). CK1943 *bkIs1943[Psnb-1::hTDP-43 WT::K4aptazyme::unc-54 3′UTR+Pmyo-3::mCherry]* was generated using a transgene with the K4 aptazyme sequence (a fusion RNA of a type III hammerhead ribozyme with a tetracycline binding RNA aptamer; Wurmthaler et al., 2019; Liachko et al., 2010) between the wild-type human TDP-43 cDNA and 3′UTR. Multicopy integrated transgenes were produced using germline microinjection, integration and outcrossing as previously described (Wurmthaler et al., 2019; Liachko et al., 2010). NLS19 *bkIs1441[Paex-3::Tau WT(4R1N)+Pmyo-2::dsRED]; bkIs1943[Psnb-1::hTDP-43 WT::K4aptazyme::unc-54 3′UTR+Pmyo-3::mCherry]* was generated by crossing CK1441 and CK1943. The CRISPR-generated whole-gene deletion CK3011 *sut-2(bk3011)* was generated as described in Kow et al. (2021). Strain NLS19 was crossed with CK3011 *sut-2(bk3011)* to generate NLS23 *sut-2(bk3011); bkIs1441[Paex-3::Tau WT(4R1N)+Pmyo-2::dsRED]; bkIs1943[Psnb-1::hTDP-43 WT::K4aptazyme::unc-54 3′UTR+Pmyo-3::mCherry]*, NLS24 *sut-2(bk3011); bkIs1943[Psnb-1::hTDP-43 WT::K4aptazyme::unc-54 3′UTR+Pmyo-3::mCherry]* and NLS25 *sut-2(bk3011); bkIs1441[Paex-3::Tau WT(4R1N)+Pmyo-2::dsRED]*. Strains with GFP-marked serotonergic neurons were generated by crossing the reporter strain JPS617 [*Ptph-1*::GFP] with CK1441, CK1943 and NLS19. Strains with GFP marked dopaminergic neurons were generated by crossing the reporter strain WG1 [*Pdat-1*::GFP] with CK1441, CK1943 and NLS19. Strains with GFP marked glutamatergic neurons were generated by crossing the reporter strain OH10972 [*Peat-4*::GFP] with CK1441, CK1943 and NLS19. Strains with GFP-marked cholinergic neurons were generated by crossing the reporter strain LX929 [*Punc-17*::GFP] with CK1441, CK1943 and NLS19. Strains with GFP-marked GABA-ergic motor neurons were generated by crossing *sut-2(bk3011)* with CK1441, CK1943, NLS19 and the reporter strain EG1285 [*Punc-47*::GFP]. See Table S1 for complete list and descriptions of all strains used in this study.

### Motility assays

Unstimulated activity and thrashing behaviors were assessed using the WormLab system (MBF Bioscience). For unstimulated activity assays, stage-matched day 1 adult *C. elegans* were transferred to 35 mm NGM assay plates seeded with 20 μl OP-50 bacteria. Animals were allowed to acclimate to conditions on assay plates at room temperature for at least 30 min before recording movements for 1 min at 7.5 frames per second. For thrashing assays, staged-matched day 1 adult *C. elegans* were given 30 min to acclimate to the assay room conditions. Approximately 50 animals were then transferred to a 35 mm assay plate by way of washing in 1 ml M9 buffer, given 1 min to standardize swimming behavior, followed by a 1 min recording time at 14 frames per second. Tracks were verified and repaired as needed. Figures show results from at least three independent replicates.

### Mechanosensation assay

Mechanosensation assays were adapted from Miyasaka et al. (2005). In brief, an eyelash was gently touched across the anterior and posterior of each day 1 adult worm successively five times each, for a total of ten touches per trial. Touch responses were summarized on a 0-2 scale (2, normal response; 1, abnormal response; 0, no response). Fifteen worms of each strain were scored per independent replicate for a total of 45 worms per genotype. Scoring was conducted blinded to genotype. Figures show results from three independent replicates.

### EPG assay

EPG comparisons were performed using the NemaMetrix ScreenChip System and associated software packages NemAquire and NemAnalysis, and Microsoft Excel. Stage-matched day 1 adult animals were washed in M9 and then incubated at room temperature in 10 mM Serotonin in M9 buffer for 20 min to induce pharyngeal pumping. Animals were loaded into a ScreenChip20 cartridge under vacuum. The electrical signal (which is driven primarily by pharyngeal pumping) was recorded individually for 2 min. Average pump frequency and duration readouts from NemAnalysis were averaged over three independent replicates per strain.

### Neurodegeneration assays

Animals were grown to day 1 or day 4 adult. Living animals were immobilized on a 2% agarose pad with 0.01% sodium azide, and intact neurons were scored under fluorescence microscopy on a DeltaVision Elite (GE) imaging system using an Olympus 60× oil objective. Scoring was conducted blinded to genotype. Figures show results from at least three independent replicates.

### Immunoblotting and quantitation

Approximately 10,000 stage-matched day 1 adult *C. elegans* were harvested and snap frozen per sample. Protein was extracted by resuspending pellets in 1× sample buffer, three sessions of 10 s sonication with cooling on ice water in between sessions, and 10 min boiling. Samples were loaded and resolved on precast 4-15% gradient SDS-PAGE gels and transferred to PVDF membrane as recommended by the manufacturer (Bio-Rad). On immunoblots, human TDP-43 was detected by a monoclonal antibody, anti-TDP-43 (ab57105[2E2-D3], Abcam, 1:10,000). TDP-43 phosphorylated at pS409/S410 was detected by a monoclonal antibody, anti-phospho TDP-43 (pS409/410, TIP-PTD-M01, Cosmobio, 1:667). Human tau was detected by a polyclonal antibody anti-tau (A0024, Dako, 1:200,000). Phosphorylated tau was detected by a monoclonal antibody anti-phosphorylated tau (AT8/PHF-Tau, MN1020, Thermo Fisher Scientific, 1:1000). *C. elegans* β-tubulin levels were measured using monoclonal antibody E7 (Developmental Studies Hybridoma Bank, 1:5000) as a loading control. HRP-labeled goat anti-mouse IgG secondary antibody (Jackson ImmunoResearch, 115-005-003) was used at a dilution of 1:2500. HRP-labeled mouse anti-rabbit IgG secondary antibody (Jackson ImmunoResearch, 211-005-109) was used at a dilution of 1:10,000. Quantitation was completed by ImageJ software densitometry analysis of scanned film images.

### Statistical analyses

All statistical analyses were performed using GraphPad Prism statistical software. Statistical significance was determined using one-way ANOVA with Tukey's multiple-comparison test. Behavioral assays are graphed using violin plots showing the distribution and density of measures of worm movement in response to the particular stimulation for each assay; all other data are presented using standard bar graphs. Error bars represent standard error of the mean (s.e.m.).

## Supplementary Material

Supplementary information
