# Supplementary material for: TDP-43 promotes tau accumulation and selective neurotoxicity in bigenic Caenorhabditis elegans
Source: Dis Model Mech. 2022 Apr 27;15(4):dmm049323. doi: 10.1242/dmm.049323 (PMC9066518; doi:10.1242/dmm.049323)
Supplement: Supplementary information [file dmm-15-049323-s1.pdf]

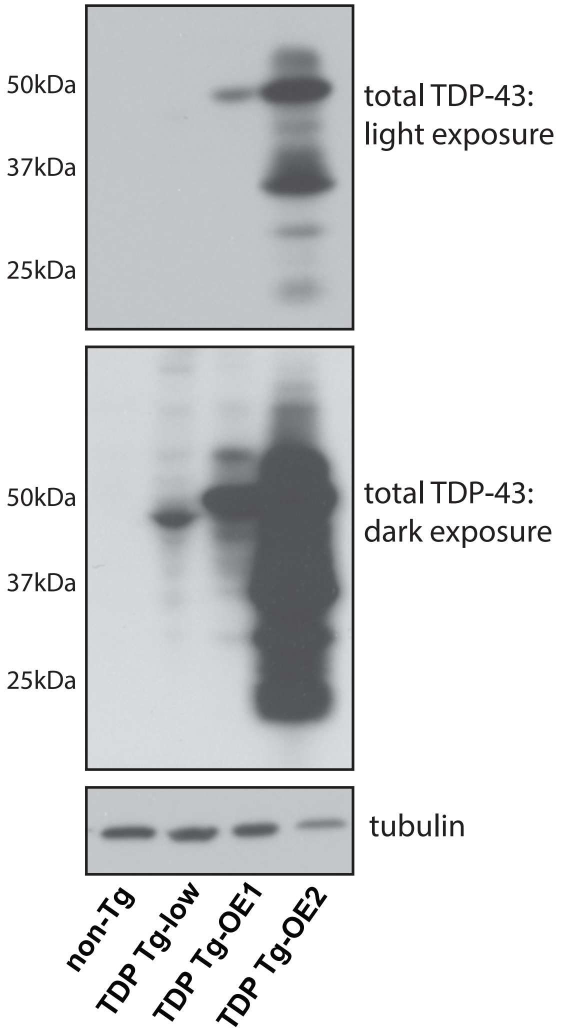


**Fig. S1. TDP Tg-low animals express low levels of TDP-43.** Immunoblot comparing TDP-43 protein levels among non-transgenic (non-Tg), TDP Tg-low, a TDP-43 transgenic line with moderate overexpression (TDP Tg-OE1), and a TDP-43 transgenic line with high levels of overexpression (TDP Tg-OE2). The TDP-43 sequence in each strain was verified by sequencing to code for full-length wild-type TDP-43. The slightly lower molecular weight of TDP Tg-low may be due to a lack of post-translational modifications, particularly phosphorylated TDP-43, which are present in the higher copy TDP-43 strains.

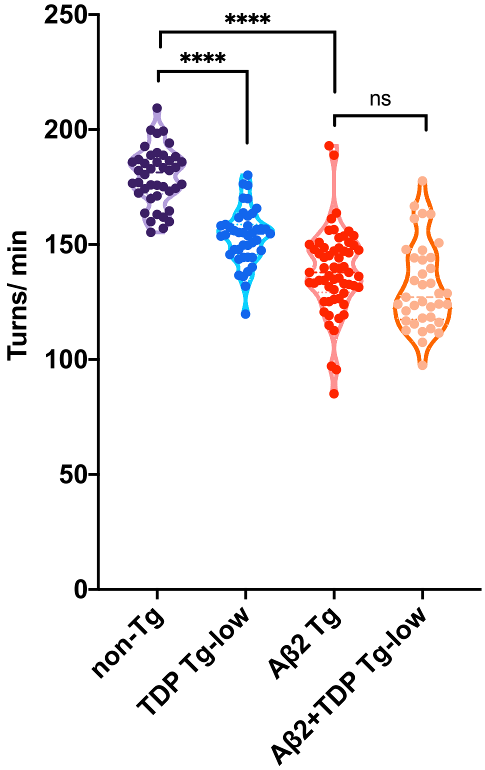


**Fig. S2. tau+TDP Tg-low animals do not synergize with Aβ.** Rates of thrashing in liquid were measured using unbiased computer-assisted tracking and analysis. The number of turns (thrashes) per second were recorded (turns/ sec). ns=not significant, ****$p<0.0001$. N=128 (non-TG), 202 (TDP Tg-low), 140 (Aβ Tg), 97 (Aβ+TDP Tg-low), from 3 independent replicates. ****$p < 0.0001$, ns=not significant.

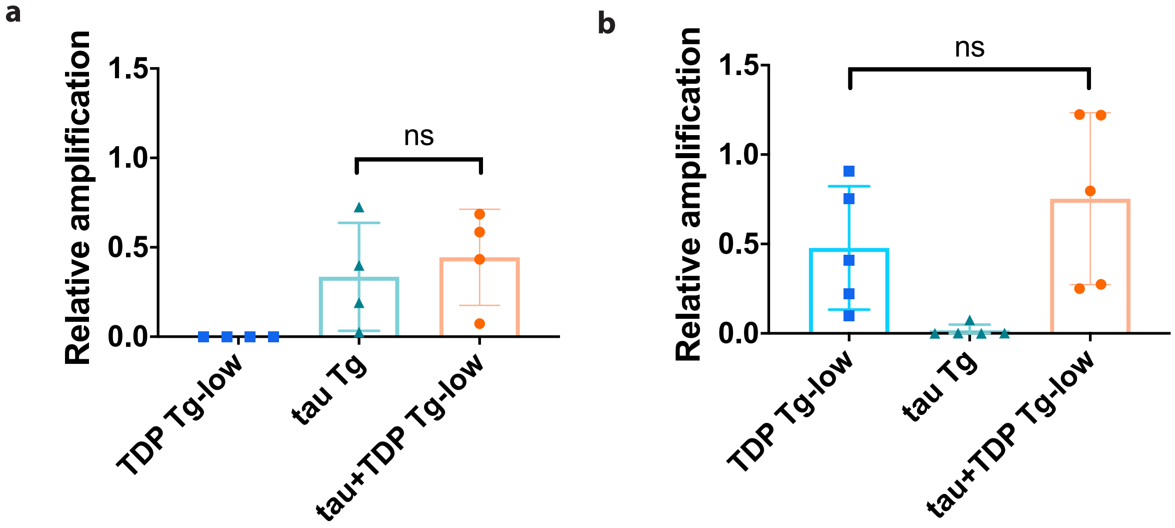


**Fig. S3. mRNA expression of tau and TDP-43 transgenes are unchanged in tau +TDP-43 Tg-low. a** Quantitative reverse-transcription PCR (qRT-PCR) testing expression of the tau transgene. tau signal is normalized to expression of an internal control gene, *rpl-32*, and plotted as arbitrary units. **b** qRT-PCR testing expression of the TDP-43 transgene. TDP-43 signal is normalized to expression of an internal control gene, *rpl-32*, and plotted as arbitrary units. ns=not significant.

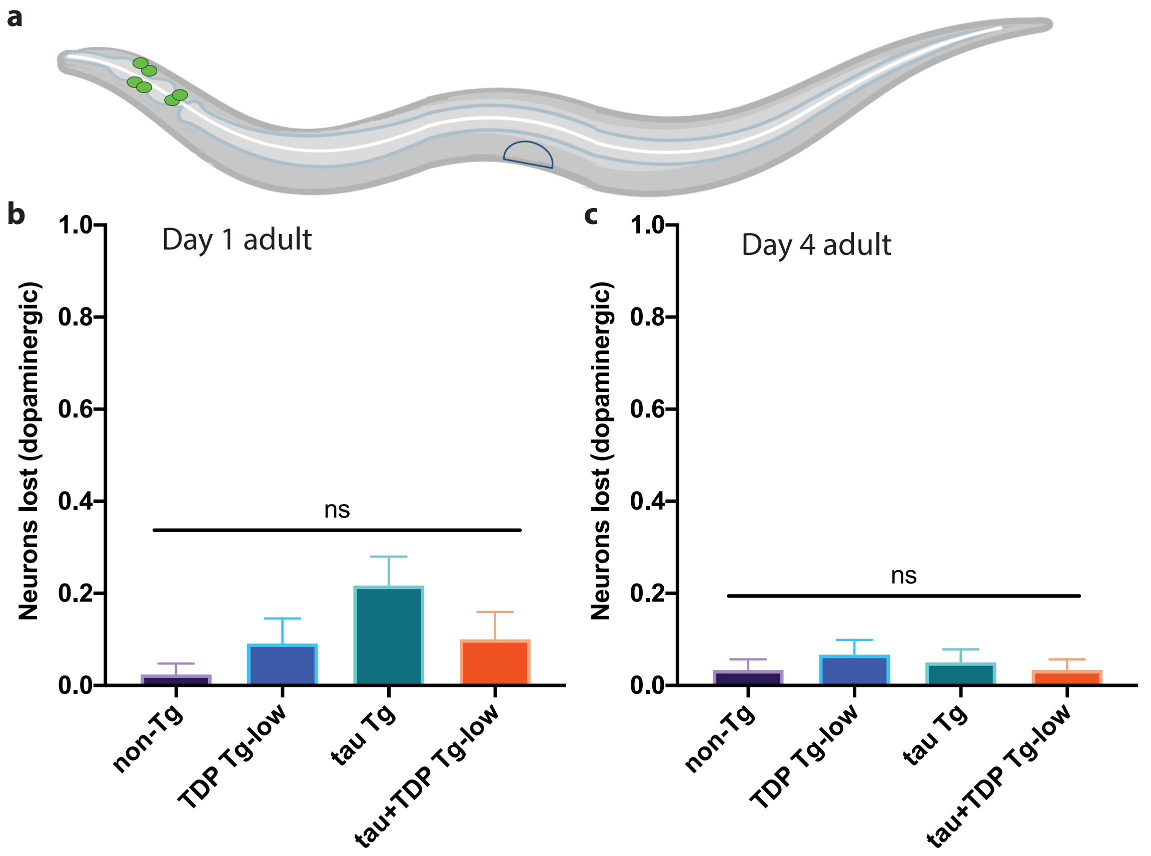


**Fig. S4. Co-expression of tau and TDP-43 does not impact dopaminergic neurons. a-c** Assessment of dopaminergic neurons in tau+TDP Tg-low animals. **a** Depiction of neurons scored (green). **b** Quantification of neurons lost in developmentally synchronized day 1 adult (**b**) and day 4 adult (**c**) *C. elegans*. ns = not significant. N>45 for all strains and timepoints scored, from at least 3 independent replicates.

Disease Models & Mechanisms • Supplementary information

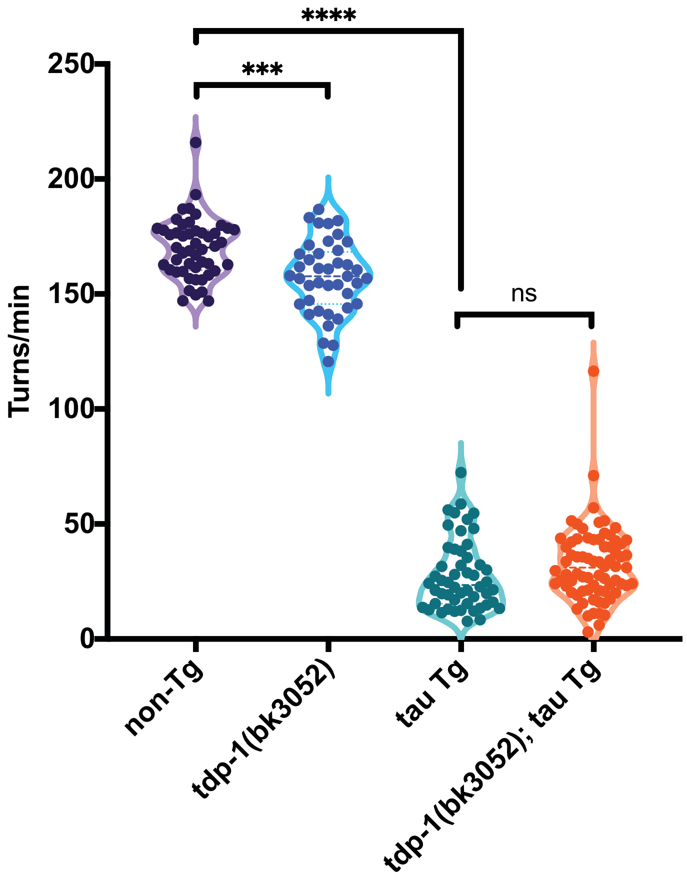


**Fig. S5. Loss of the *C. elegans* homolog of TDP-43, *tdp-1*, does not alter tau-driven motility dysfunction.** Rates of thrashing were measured using unbiased computer-assisted tracking and analysis. The number of turns (thrashes) per minute were recorded (turns/ min). ns=not significant, ***P<0.001, ****p<0.0001. N=49 (non-TG), 41 (*tdp-1(bk3052)*), 52 (tau Tg), 73 (*tdp-1(bk3052)*; tau Tg), from 3 independent replicates.

Disease Models & Mechanisms • Supplementary information

## Table S1. Strains used in this study.

| Abbreviation | Strain name | Genotype | Transgene |
|---|---|---|---|
| non-Tg | N2 | wild-type | |
| tau Tg | CK1441 | *bkIs1441[Paex-3::Tau WT(4R1N)+Pmyo-2::dsRED]* | human tau |
| Aβ Tg | GRU102 | *gnaIs2[Pmyo-2::YFP+Punc-119::Abeta1-42]* | human Aβ |
| Aβ Tg 2 | CL2355 | *dvIs50[pCL45(snb-1::Abeta1-42::3'UTR(long) + mlt-2::GFP]* | human Aβ |
| polyQ Tg | CK241 | *bkIs241[pF25B5.3::Q86-YFP]* | 86 repeats of glutamine |
| TDP Tg-low | CK1943 | *[Psnb-1::hTDP-43 WT::K4aptazyme::unc-54 3'UTR+Pmyo-3::mCherry]* | human TDP-43 |
| tau+TDP Tg-low | NLS19 | *bkIs1441[Paex-3::Tau WT(4R1N)+Pmyo-2::dsRED]; bkIs1943[Psnb-1::hTDP-43 WT::K4aptazyme::unc-54 3'UTR+Pmyo-3::mCherry]* | human tau and TDP-43 |
| *sut-2(-);* tau+TDP Tg-low | NLS23 | *sut-2(bk3011); bkIs1441[Paex-3::Tau WT(4R1N)+Pmyo-2::dsRED]; bkIs1943[Psnb-1::hTDP-43 WT::K4aptazyme::unc-54 3'UTR+Pmyo-3::mCherry]* | human tau and TDP-43 |
| *sut-2(-);* TDP Tg-low | NLS24 | *sut-2(bk3011); bkIs1943[Psnb-1::hTDP-43 WT::K4aptazyme::unc-54 3'UTR+Pmyo-3::mCherry]* | human TDP-43 |
| *sut-2(-);* tau Tg | NLS25 | *sut-2(bk3011); bkIs1441[Paex-3::Tau WT(4R1N)+Pmyo-2::dsRED]* | human tau |
| | EG1285 | *oxIs12*[*Punc-47*::GFP + *lin-15(+)*] | GABAergic neuron GFP |
| | JPS617 | *Ptph-1*::GFP | serotonergic neuron GFP |
| | WG1 | *Pdat-1*::GFP | dopaminergic neuron GFP |
| | OH10972 | *Peat-4*::GFP | glutamatergic neuron GFP |
| | LX929 | *Punc-17*::GFP | cholinergic neuron GFP |
| | CK3052 | *tdp-1(bk3052)* | |

Disease Models & Mechanisms • Supplementary information

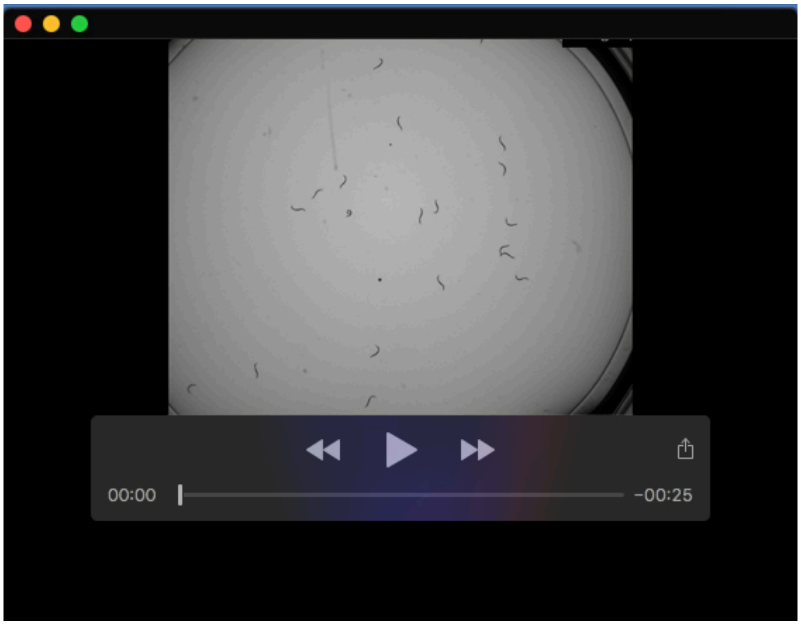


**Movie 1. Non-Tg animals have normal swimming in liquid.**

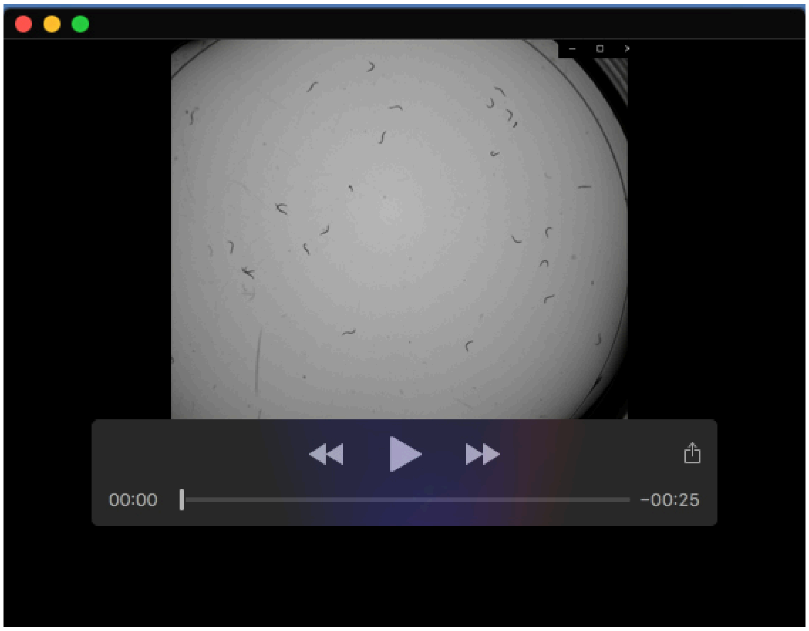


**Movie 2. TDP Tg-low animals have decreased rates of swimming in liquid.**

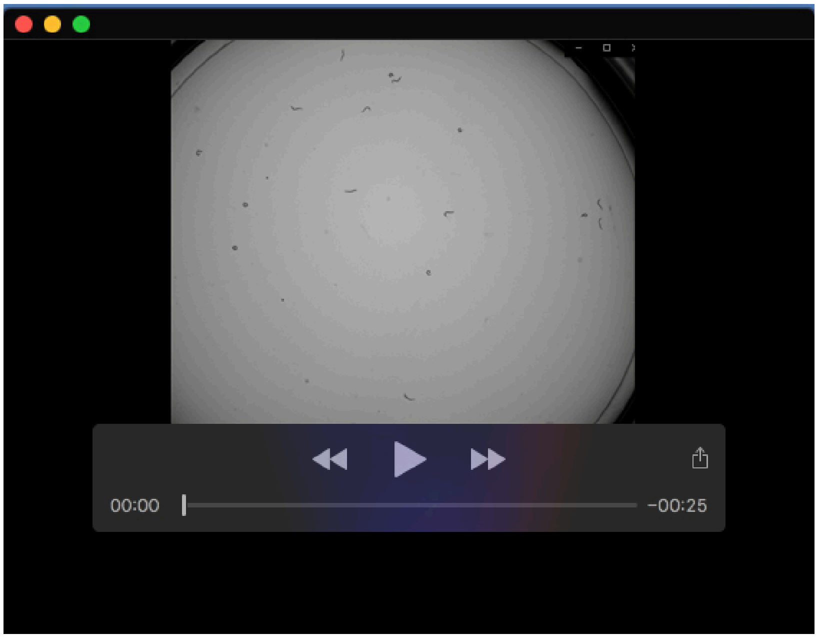


**Movie 3. tau Tg animals have significantly impaired swimming in liquid.**

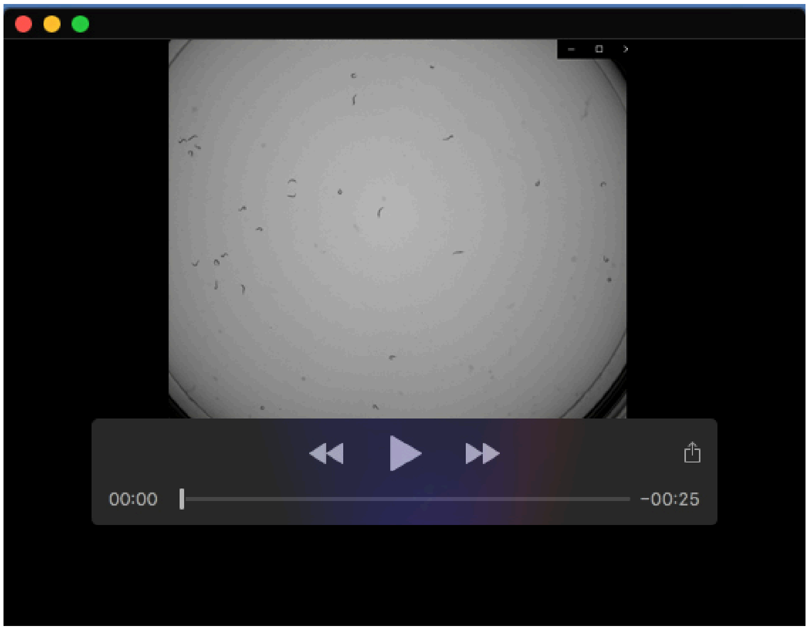


**Movie 4. tau+TDP Tg-low animals are unable to swim in liquid.**

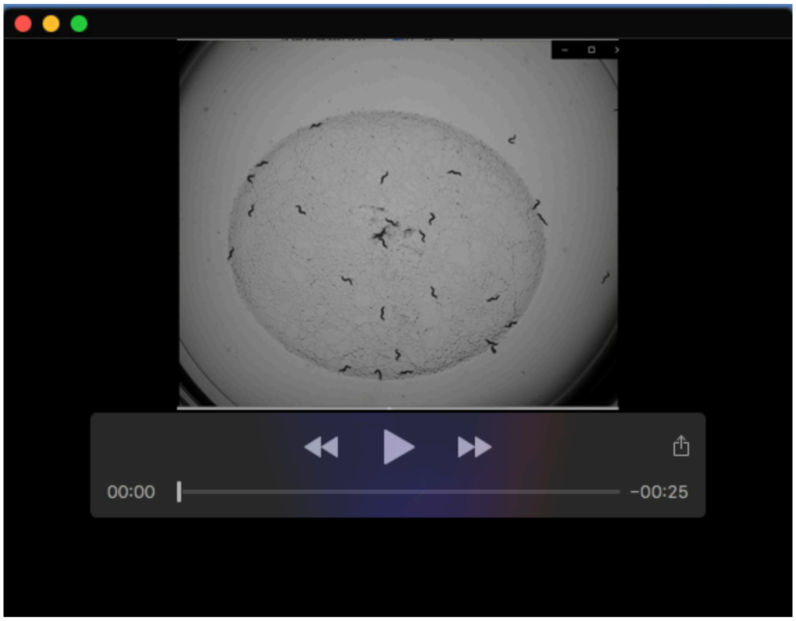


Movie 5. Non-Tg animals have normal motility on a solid surface.

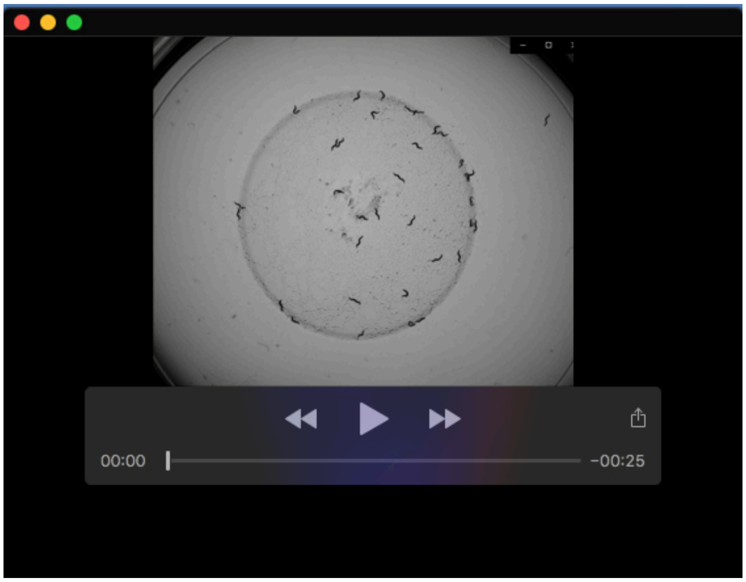


Movie 6. TDP Tg-low animals have slower motility on a solid surface.

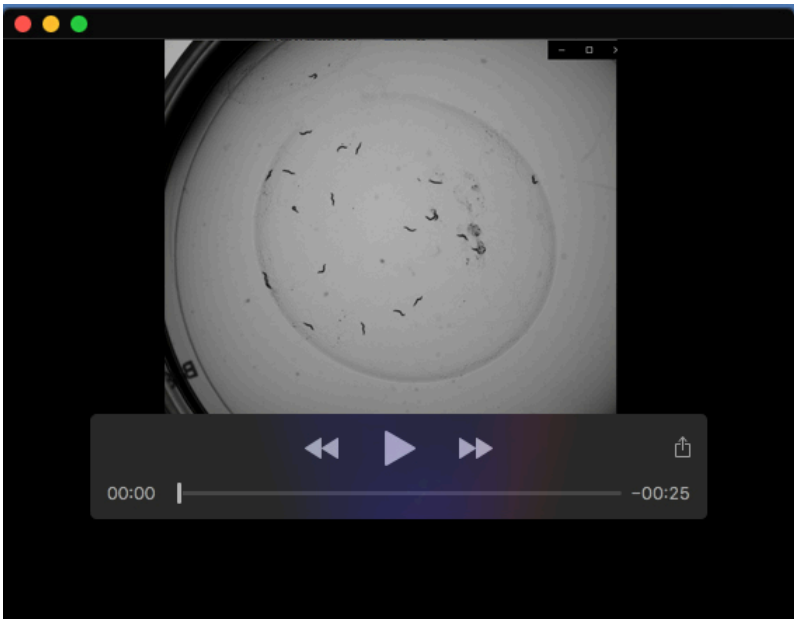


**Movie 7. tau Tg animals have impaired motility on a solid surface.**

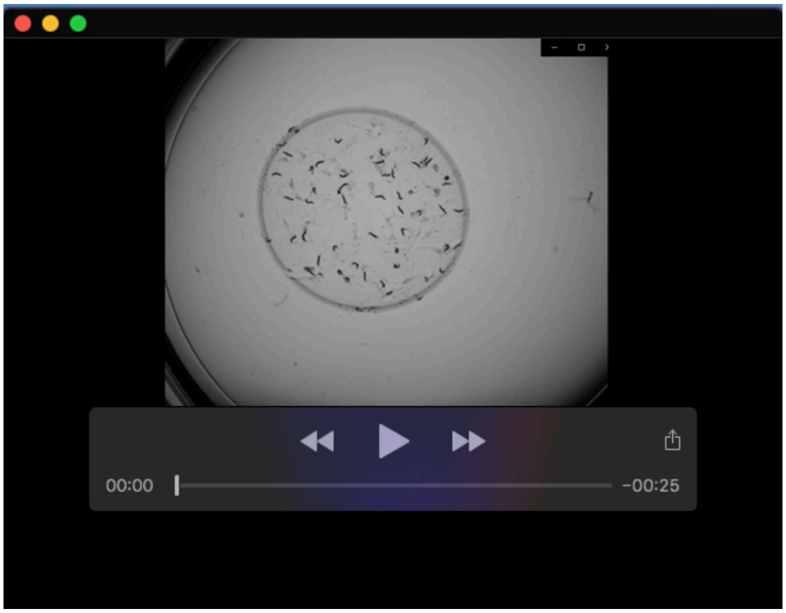


**Movie 8. tau+TDP Tg-low animals have significantly impaired motility on a solid surface.**
